# Progress in State Medicine

**Published:** 1897-06-05

**Authors:** 


### Progress in State Medicine.

(*Continued from page* 32).

**School Hygiene.**—Shirley-Murphy[1] shows that the prevalence of both scarlet fever and diphtheria is markedly influenced by school attendance, there being a sudden drop as soon as the holidays commence. This fall in the prevalence curve begins a week sooner in scarlet fever than in diphtheria, and in the former extends to children above the school age, whereas in diphtheria the depression extends chiefly to infants, and in them begins a fortnight later than in those attending school. Were systematic medical inspection of our schools conducted as in America it would no doubt result in the early detection of many cases of infectious disease, and consequent diminution of the spread of infection. As[2] a result of the first day's work of the New York inspectors, 140 children were found unfit for association with their school-mates. Of these, 14 were cases of diphtheria, 3 of measles, 1 of scarlet-fever, and 3 of mumps. In addition, 35 cases of contagious eye disease and 55 cases of tinea were isolated.

The reports of the *British Medical Journal* on the hygienic condition and administration of metropolitan district and separate schools accentuates the evils, both moral and physical, resulting from the aggregation of children in barrack schools. Ophthalmia, ringworm, eczema, and other contagious disorders are rife, and epidemics of scarlet fever, whooping-cough, and measles are of frequent occurrence. At the Sutton school it is calculated that each child was ill seven times during the year, and at one time 16 per cent. of the scholars required isolation, and 30 per cent. were disabled by sickness of various kinds. Ophthalmia is regarded as a disease created in the barrack schools, a direct consequence of overcrowding, imperfect ventilation, and bad washing accommodation. At Hackney the children did not leave the school precincts for two or three years at a time, and generally the laws of health were neglected. Guardians too frequently altogether ignored the recommendations of the school inspectors, and so cumbersome are the methods of procedure necessary to enforce obedience that the Local Government Board is practically powerless. The teaching in these institutions is defective, and the standard lower than that in elementary schools. The children are dull, do not play, are self-indifferent, and grow up ignorant of worldly and domestic affairs. Whilst the cost of keep is excessive, the schools are understaffed. The children need greater variety of treatment, more individual care, and more natural, home-like lives. Where the inmates have been sent to outside schools, and allowed to mix with other children, and where it has been possible to send them into the country for holidays, a marked improvement has been manifested. The village community system, as partially adopted at Hornchurch, is warmly commended, and the effect of thus breaking up the children into groups is very marked, the sickness rate in this particular community being 8 per cent. as compared with 30 per cent. at the neighbouring barrack school at Hanwell.

**Industrial Hygiene.**—The hygienic and social conditions under which factory life is carried on in Germany are in many respects superior to these in this country.[3] Under the system of compulsory insurance of the working classes 7,760,000 persons are protected in case of sickness, 18,192,000 for accident, and 11,510,000 in case of invalidity (and old age). In Bavaria the notification of cases of industrial lead, mercury, and phosphorus poisoning has led to a marked declension in the number of people incapacitated from work from these causes. The hours of labour, however, are longer than in this country, and the child worker is very imperfectly protected. The Industrial Code of 1891 has put an end to Sunday labour, and has limited the number of hours for women to 11, a length of time still in excess of that allowed in England. The order of the Home Secretary,[4] insisting upon the use of the higher explosives in coal mines, and the abolition of the use of gunpowder, should result in a considerable saving of life amongst miners. Whilst this class form 1-36th of the population of our country, they account for 1-5th of the fatal accidents that occur in all industries, the deaths during 1895 numbering 1,096. Generally the death-rate amongst miners is double that of those who work on the surface. The flame created, and heat generated by the explosion of gunpowder is especially likely to give rise to accident in dry pits, where the coal dust is in a state of very fine division, for in these pits precautions are more likely to be neglected than where the mine is known to be fiery—that is, to contain some considerable quantity of fire damp. Roburite and tonite are the new explosives most commonly employed, and whatever objections may be raised to them on the score of increased expense and excessive blasting force, the complaint that the fumes give rise to peculiarly evil effects cannot be maintained, for nothing of the kind has been observed in the Welsh collieries, where they have been long in use. Under the Coal Mines Act the provision of surgical dressings and appliances is rendered compulsory, but no such requirement is essential under the Factories Act, although in many cases the workers are exposed to an equal risk of injury. Recent legislation in Austria[5] compels all manufacturing establishments to keep appliances and dressings for wounds and for the arrest of hæmorrhage constantly in readiness, and it is to be hoped that a similar arrangement may be adopted in this country with a consequent saving of life and suffering.

Snell[6] draws attention to the peculiar form of sickness which attacks men working in compressed air. Although no fatal case occurred amongst the workmen during the construction of the Blackwall Tunnel, yet the use of compressed air has been responsible for a by no means inconsiderable number of deaths. The most common symptoms are pains and paralysis in the limbs, auditory vertigo, and epistaxis, and these, the author thinks, depend chiefly on an increased solution by the blood of the gases of the compressed air, and the liberation of these on the pressure being removed. In a lesser degree exhaustion, carbonic acid poisoning, and congestion of viscera may play a part in the causation.

**Refuse Disposal.**—Fyfe[7] calculates that ashpit refuse averages five hundredweight per head per annum, or for a town of 10,000 inhabitants six and a half tons per diem. This amount can easily be burned in a two-cell destructor in 24 hours, the average temperature being 1,350 deg. Fahr. To prevent the escape of noxious fumes, and to secure a proper draught, it is recommended that the chimney shaft be 120 ft. high. With such a furnace sufficient heat is generated to keep a 12 horse-power engine going for 24 hours, the power being utilised for pumping, lighting, &c. The work of cremation cannot at present be conducted at a profit, but it is hoped that with boilers and furnaces of an improved construction this end may be reached, especially if some means of storing heat be discovered. Tipping rubbish, or using it as a coarse manure after sorting, are useless and expensive methods. Chiapponi[8] has made a detailed examination of dust destructors in this country and upon the Continent. The types used at Ealing, Newcastle, Liverpool, and Edinburgh are described in detail. The Manchester cremator is spoken of as being extremely simple and effective, and that at Leeds, of the Horsfall type, with steam draught, is described as securing almost perfect combustion. Such apparatus is but little used on the Continent, Brussels being the first town to adopt the English methods. Cremation of refuse has not been found a success in Berlin, but this appears to be due to the nature of the material collected. Chiapponi, after detailing the various methods in which the heat generated may be applied, and describing the utilisation of the clinker as cement, paving-stone, or road material, extends a hearty support to the process of refuse cremation as being economical and labour-saving, and a very great source of safety during epidemics. In direct contradiction to the above is the finding of the committee appointed by the Paris Academie de Médicine.[9] Their investigations have extended over a period of more than ten years, and they now report in favour of distribution and removal of garbage as against cremation, the former process being less costly, more hygienic, and most beneficial to agriculture.

[1] Annual Report to London County Council, 1895. [2] Med. News of New York, April 10, 1897. [3] B. M. Journal, Feb. 13, 1897. [4] Legislation and the Safety of the Coal Miner, Ibid. [5] Deutsch Med. Woch, Dec. 17, 1896. [6] Compressed Air Illness, H. K. Lewis. [7] Notes on the Utilisation of Town Refuse, Sanitary Record, Oct. 23, 1896. [8] Sull elimenazione—delle Spazzature, Giornale dilla Reale Società Italiana d'Igiene. [9] The Journal, Jan. 9, 1897.

